# Influence of Season and Diet on Fiber Digestion and Bacterial Community Structure in the Rumen of Muskoxen (*Ovibos moschatus*)

**DOI:** 10.3390/microorganisms6030089

**Published:** 2018-08-20

**Authors:** Emilio M. Ungerfeld, Mary Beth Leigh, Robert J. Forster, Perry S. Barboza

**Affiliations:** 1 Lethbridge Research Centre, Agriculture and Agri-Food Canada, 5403 1st Ave S, Lethbridge, AB T1J 4B1, Canada; emilio.ungerfeld@inia.cl; 2 Department of Biology and Wildlife, Institute of Arctic Biology, Fairbanks, AK 99775-7000, USA; mbleigh@alaska.edu; 3 Department of Biology and Wildlife, University of Alaska, Fairbanks, AK 99775-7000, USA

**Keywords:** muskoxen, rumen, bacterial diversity, digestion

## Abstract

We studied the relationship between fiber digestion and the composition of the bacterial community in the rumen of muskoxen at the start and the end of the annual window of plant growth from spring to fall. Eight ruminally cannulated castrated males were fed brome hay or triticale straw (69.6% vs. 84.6% neutral detergent fiber, respectively) that were similar in fiber content to the sedges consumed by wild muskoxen (64.5 to 71.7% neutral detergent fiber). Muskoxen digested fiber from both forages faster and to a greater extent when straw rather than hay was consumed. Fiber digestion was therefore inducible by diet 4 in each season. We used 16S rRNA sequences from ruminal contents to study how season and diet affected the bacterial community and how the latter related to fiber digestion. We found that Bacteroidetes and Firmicutes accounted for 90% of the sequences at the level of Phylum, which is typical for the mammal gut microbiome. Using partial least square regressions, it was found that between 48% and 72% of the variation in fiber digestion was associated with 36–43 genera of bacteria. The main fibrolytic bacteria typical of domestic ruminants were generally not among the most important bacteria associated with fiber digestion in muskoxen. This reveals that muskoxen rely upon on a large suite of bacterial genera that are largely distinct from those used by other ruminants to digest the cell walls of plants that vary widely in both abundance and nutritional quality through the year.

## 1. Introduction

In ruminant herbivores, the reticulo-rumen supports the largest microbial community in the gut, which accounts for most of the digestion of plant dry matter (DM) and fiber by bacteria, protozoa and fungi. The ruminal microbiome provides the host with a wide array of enzymes to degrade the complex cell walls and the potentially toxic compounds of plants [1,2]. The composition and size of this microbial community depends upon the physical and chemical conditions of digesta flow and buffering provided by the host through ruminal structure, motility and secretion [3]. The host animal and its rumen microbiome co-evolve to adapt to the challenges imposed by the diet available in the habitats used by an animal population [4,5,6]. The gut microbiome is influenced by the host through physicochemical conditions in the gut, endogenous secretions, and intake of foods that become substrates for microbial digestion and fermentation [3,7].

Ruminants are obliged to vary their diet with the annual cycle of temperature, light and snow that dictates the growth of plants in temperate and Arctic regions [8]. Muskoxen (*Ovibos moschatus)* consume a fibrous diet of sedges and grasses throughout their Arctic range but also include stems and leaves of woody browse in their diet at lower latitudes [9,10,11,12,13]. During the long winter, forage is scarce, and often covered by snow, which often results in fasting and mass loss of muskoxen even though appetite and metabolic rate are reduced [14,15,16]. Feed consumption in the short summer and early fall has to be rapid enough to support growth or reproduction and body mass gain for the ensuing winter. Muskoxen have therefore evolved strong hyperphagic behavior in the late summer and early fall when forage biomass is high. This increase in feed consumption is not only a consequence of greater forage availability and quality. Even if fed the same batch of grass hay all year round, muskoxen exhibited a marked increase in feed intake in the summer/fall compared to winter/spring [17,18,19]. In domestic ruminants, greater intake is generally associated with a decrease in digestibility because fractional passage rate from the reticulo-rumen increases [20]; however, in muskoxen, hyperphagia occurred without loss of digestibility, because the rate of digestion in the rumen increased for DM, C, N and fiber fractions, while ruminal retention time was conserved as digesta fill also increased [18]. Hyperphagia was accompanied by increased concentrations of short chain fatty acids (SCFA) in the rumen, which indicated an increase in microbial fermentation with food intake [18]. Increased microbial activity was also supported by close regulation of pH, SCFA and osmotic concentration of rumen fluid before and after feeding in muskoxen during autumn hyperphagia [21]. Muskoxen therefore alter ruminal function (i.e., regulation of pH and osmolarity as well as digesta flow) to support seasonal changes in microbial activity often associated with changes in food intake.

The ruminal environment for microbes in muskoxen is similar to that of other grazers including domestic cattle and sheep. Ruminal structure and function favor the stratification of digesta in muskoxen to the same extent as other grazers [22,23,24]. The morphology of the ruminal mucosa and the omasum of muskoxen are also similar to other grazers. These attributes are not simply responses to the diet but evolutionary adaptations that develop in young muskoxen before they begin consumption of plants [25]. The evolutionary history of the muskoxen may influence the diversity of its microbes. Muskoxen (genus *Ovibos* de Blainville, 1816) are most closely related to gorals (genus *Nemorhaedus* Hamilton Smith, 1827) and mountain goats (genus *Oreamnos* Rafinesque, 1817) that inhabit alpine areas and feed on both graminoids and woody browse [26,27]. Isolation and genetic bottlenecks in muskoxen may have further contributed to differentiation of the microbiome [28,29,30]. Recent analyses of sequences from fungal and protozoal messenger RNA from the rumen of muskoxen indicate that the microbiome produces novel enzymes for fiber digestion that are markedly different from those of other herbivores including domestic ruminants [31]. The composition of the bacterial community in muskoxen feces was recently reported [32,33], but has not been previously reported for the rumen.

We studied how the composition of the bacterial community in the rumen of muskoxen was influenced by diet and season to examine the functional response associated with fiber digestion when muskoxen consume low- or medium-quality forage at the beginning and the end of the annual cycle of plant growth. We hypothesized that fiber digestion would increase with the fiber content of the forage and also increase from spring to autumn when animals typically gain mass. The objective of this study was to identify fibrolytic microorganisms potentially important to muskoxen and compare that community to those organisms that have been functionally important to other herbivores especially domestic ruminants.

## 2. Materials and Methods

### 2.1. Animal Trial and Feed and Fecal Sampling

The study was conducted at the Robert G. White Large Animal Research Station, Fairbanks, AK. All procedures were approved under protocol 139821 by the Institutional Animal Care and Use Committee at the University of Alaska Fairbanks. We used 8 castrated muskoxen that were ruminally cannulated several years before this study. Muskoxen were housed in individual pens and blocked into two groups based on their initial body mass. Each group of animals was ad libitum fed triticale (*Triticosecale hexaploide*) straw as a low-quality diet or brome (*Bromus* spp.) hay as a medium-quality diet. All animals were supplemented with 335 g d^−1^ of a protein and mineral supplement (M Ration, Alaska Pet and Garden, Anchorage, AK; 14.0% crude protein (CP), 24% Neutral Detergent Fibre (NDF), 16% Acid Detergent Fiber (ADF), 2% lignin and 2% total ash, DM basis). Animals were fed the forage component of their diets at mid-morning and late afternoon, and the protein and mineral supplement at mid-morning. Fresh water or snow was always available ad libitum.

Each experimental period comprised three weeks of adaptation to diets followed by one week of measurements and sampling, which comprised the study week. After the first experimental period, the forages were switched for the second period of the trial (i.e., cross over design). Two trials were conducted, in the spring (April and May) and fall (August and September) of 2009. All the animals were fed brome hay and the mineral supplement between both trials.

Muskoxen were weighed at the beginning and end of each period. Mean body mass at the beginning of the spring trial was 268 ± 18.4 and 278 ± 27.2 kg (*p* = 0.56) for the hay and straw diet groups, respectively. Mean body mass at the beginning of the fall trial were 263 ± 25.3 and 272 ± 34.9 kg (*p* = 0.71) in the same order. The same batches of triticale straw and brome hay were used in the spring and fall trials. One of the animals was removed from the experiment in the fall trial due to an infection of the horn boss.

All measurements and sampling took place during the study week before the morning feeding. Weekly feed intake was measured in the fall by weighing the forage supplied every day during the study week and subtracting the refusal that was left at the end of the week to calculate the average daily intake for the study week. Feed samples were taken every day during the study week, as well as refusal samples at the end of the week. Fresh fecal samples were obtained for determination of digestibility. All samples of feed, refusals and feces were stored frozen for analysis.

### 2.2. In Situ Digestibility

Triticale straw and brome hay were ground through a 1.27 mm screen (Wiley mill, A.H. Thomas, Philadelphia, PA, USA) for incubation within a polyester bag (50-µm pore size; Ankom Technology, Macedon, NY, USA) to measure digestion kinetics in situ within the rumen [18,34]. Each polyester bag contained 0.75 g (fresh mass) of each forage. Prior to incubation in the rumen, the bags and the substrate were dried to constant mass at 55 °C. Bags were simultaneously inserted into the rumen before the morning feeding of the first day of the study week and removed at 24, 36, 48, 72, 96 and 120 h. Control bags without substrate were also placed in the rumen and removed at 120 h. Digestion bags were stored at 4 °C until they were rinsed with tap water for a few seconds and dried to constant mass at 55 °C for subsequent analysis. Sealed dry bags without sample were weighed as controls before and after incubation to measure mass gain of the bag. Net content of dry sample was calculated by subtracting the dry mass of the bag before and after incubation from the gross mass.

### 2.3. Ruminal pH and Sampling

On days 1 and 5 of the study week, ruminal pH was measured (Oakton Instruments pHmeter, pH6 Acorn Series, Vernon Hills, IL, USA) by inserting an electrode (Premium Gel Glass Combo Cat # 476566 electrode, Corning, NY, USA) into the rumen through a PVC tube.

On day 4 of the study week, contents from different parts of the rumen were sampled using forceps and mixed together in a beaker. The solids were separated from the liquid fraction of the ruminal contents using a coffee press (Bodum Inc., Triengen, Switzerland). Three to four grams of solids were then combined with 5 mL of grinding buffer (100 mm pH 8 EDTA, 100 mm pH 8 Tris, 1.5 mm NaCl) containing 1 g L^−1^ of Proteinase K (Sigma, Oakville, ON, USA). The mixture of ruminal solids and grinding buffer was then wrapped in aluminum foil and immediately frozen in liquid nitrogen [35]. Frozen ruminal samples were transported on dry ice to the laboratory, where they were stored at −80 °C until processed.

### 2.4. Chemical Analysis

Samples of feed, feces and the in situ bags were dried to constant weight at 55 °C for 3 day to determine DM content. Analyses of N, fiber and ash followed the procedures described in Peltier et al. [18] and Barboza et al. [17]. Ash content was subtracted from neutral and acid detergent fiber to obtain ash-free fractions (reported as NDF and ADF, respectively). Cellulose was calculated as the difference between ADF and ash free-lignin, and hemicellulose as the difference between NDF and ADF [18].

### 2.5. Overall Tract Apparent Digestibility Calculation

Overall tract apparent digestibility was calculated using lignin as an indigestible marker [18]:

$$\text{Digestibility }(\text{kg }{\text{kg}}^{-1})\;=\;1\;-\;(\frac{\mathrm{nutrient}}{\mathrm{lignin}})\;\mathrm{feces}\;\times \;(\frac{\mathrm{nutrient}}{\mathrm{lignin}})\;{\text{diet}}^{-1}$$

We obtained the nutrient to lignin ratio in the diet as the average of the nutrient to lignin ratio in the forage (for) and in the protein and mineral supplement (suppl), weighted by their respective DM consumptions:

$$(\frac{\mathrm{nutrient}}{\mathrm{lignin}})\;\mathrm{diet}\;=\;[(\frac{\mathrm{nutrient}}{\mathrm{lignin}})\;\mathrm{for}\;\times \;\mathrm{for}\;\mathrm{DMI}+(\frac{\mathrm{nutrient}}{\mathrm{lignin}})\;\mathrm{suppl}\times \mathrm{suppl}\;\mathrm{DMI}]÷(\mathrm{total}\;\mathrm{DMI})$$

We did not measure forage intake in the spring and were therefore unable to determine digestibility in the spring.

### 2.6. DNA Extraction from Ruminal Solids

DNA was extracted from the solid fraction of ruminal digesta using the procedure of Kong et al. [35]. Briefly, ruminal solids were manually ground in liquid nitrogen to a fine powder. After combining with proteinase K (1 mg/mL; Sigma-Aldrich Canada Ltd., Oakville, ON, Canada), the samples were further ground for 5 min in liquid nitrogen using a Retsch RM100 grinder (Retsch GmbH, Haan, Germany) with a pre-chilled grinding chamber. The sample (15 mL) was mixed with 1.5 mL of 20% SDS, incubated at 65 °C for 45 min, and then centrifuged at 19,200 *g* at room temperature for 10 min. Supernatants were combined with an equal volume of a sterile 2% agarose, poured into Petri plates and allowed to solidify.

Agarose containing DNA was cut from the Petri plates and equilibrated in TBEG buffer (100 mL/L 5 × TBE buffer and 500 mL/L pure glycerol). Samples were stored in TBEG buffer at 4 °C. DNA was isolated from the agarose using the QIAquick Gel Extraction Kit (Quiagen Inc., Mississauga, ON, Canada).

### 2.7. Sequence Analysis

DNA concentration was quantified with a Synergy HT Platereader (Fisher Scientific, Ottawa, ON, Canada) using a Quant-it PicoGreen dsDNA Assay Kit (Invitrogen, Burlington, ON, Canada), and sent to the Research and Testing Laboratory (Lubbock, TX, USA), where pyrosequencing was carried out using the bTEFAP FLX massively parallel method [36]. A 100-ng DNA aliquot was used as a template in a 50-µL PCR reaction. The 16S rRNA gene universal bacterial primers 28F [37] and 519R [38] were used to obtain a 450-bp amplicon. The rest of the pyrosequencing procedure has been described by Dowd et al. [36]. The metadata and sequence reads are available at the European Nucleotide Archive (http://www.ebi.ac.uk/ena) under study accession number PRJEB6760 and run accession numbers ERS1280392-ERS1280421.

FASTA sequences were processed using mother v 1.12.3 [39]. Sequences were trimmed to a maximum of 450 bases. We removed ambiguous sequences with more than 8 homopolymers or less than 150 bp. The sequences were then aligned against the Silva v 108 REF database [40], which was modified to contain a greater representation of high quality rumen-originating sequences. The sequences were screened and filtered to overlap the same sequence space and then clustered to group sequences that differed by only 2 base pairs using the pre.cluster command. Chimeric sequences were then removed using chimera.slayer when tested against the silva.gold database. Chao and Shannon indexes of diversity were calculated by the software.

The resulting FASTA file for each sample was classified using mother against a custom Silva_108 REF database, modified to contain high-quality rumen sequences and annotated to include node designations for uncultured clusters. These clusters were named after the clone first submitted to Genbank in each cluster. Percentages of sequences in each sample at each classification level were calculated using a custom perl summation script.

Sequences were classified according to the number of shared branching points. We used a maximum of 7 branching points to assign each sequence to the lowest taxonomic level from phylum to species. Almost all groups in higher order clades (e.g., phylum) in the database corresponded to well-identified taxonomic groups. However, lower-order clades (e.g., species) included a greater proportion of unclassified groups.

This initial database of bacterial sequences was censored to remove rare sequences (i.e., present in one or two samples) that biased subsequent analyses of diversity and function (i.e., fiber digestion kinetics). The sequence database was trimmed by calculating Cook’s influence distances for NDF digestion rate against 16S rRNA gene sequence proportion in every taxonomical clade. Those sequences with significant Cook influence distances (i.e., >F_2,28,0.05_) were eliminated.

### 2.8. Statistical Analysis

We examined the effects of diet (fixed effect), season (repeated measures fixed effect) and their interaction on changes in body mass, ruminal pH, kinetics of fiber digestion (see calculations described below) and bacterial populations. Models also included the fixed effect of sequence (hay first or straw first) and the random effect of the animal nested in sequence. Because DM intake in the spring was not measured, DM intake and overall tract digestibility were analyzed only for the fall.

Fiber digestion was parametrized for each combination of animal-season-diet-substrate for digestion rate and extent. We fitted least squares negative exponential regression equations to the digested residue (D) of NDF, cellulose and hemicellulose over time:

D = a + b × (1 − exp^−c × t^)

where t is time (h) and c is fractional digestion rate. Predicted digestion at 120 h (%) was calculated from each parametrized regression equation. Subsequently, fractional digestion rate and predicted digestion at 120 h for the NDF, cellulose and hemicellulose fractions were modeled as a function of season, substrate, diet, animal and sequence as previously explained.

Neutral detergent fiber, cellulose and hemicellulose fractional digestion rate and predicted digestion at 120 h were regressed against ruminal pH, substrate, and their interaction. Results are reported as partial R^2^ (if significant) and the *p* value for the regression coefficient.

We examined the effect of season, diet and their interaction on the 16S rRNA gene proportion of bacterial phyla with a MANOVA multivariate model. Effects of season, diet and their interaction on bacterial 16S rRNA gene proportions was further analyzed through a univariate ANOVA on the 16S rRNA gene proportion of each bacterial phyla separately. Results are expressed as mean ± standard error with corresponding *p* values.

The association between all bacterial 16S rRNA gene sequence proportions and all 6 fiber digestion parameters (NDF, cellulose and hemicellulose digestion rate and extent at 120 h) was studied using partial least square regressions fitted separately for the hay and straw substrates. Firstly, taxonomic clades—phyla, classes, orders, families, genera and species—were compared for their simultaneous prediction of all 6 fiber digestion kinetics variables. For both the hay and straw substrates, the best predicting clade in terms of the percentage of variation in fiber digestion kinetics explained was genus, and subsequently the relationships of each NDF, cellulose and hemicellulose digestion rate and extent in hay and straw with the 16S rRNA gene proportion of bacterial genera were analyzed. In all partial least squares regressions analyses, the number of latent vectors was selected through one at a time-cross validation. All variables were standardized (centered and scaled).

All statistical analyses were conducted using JMP 8.0.2 [41]. All analyses that included the random effect of animal nested in sequence were fitted using a restricted maximum likelihood algorithm. Non-significant (*p* > 0.10) interactions were eliminated. If interactions were present (*p* < 0.10), the main effects of one factor were described by comparing them across the levels of the other factors in the interaction.

## 3. Results

### 3.1. Feed Composition

Feed composition is presented in Table 1. Straw contained more NDF (*p* < 0.001), ADF (*p* < 0.001), and cellulose (*p* < 0.001) than hay, but there was no difference in lignin content (*p* = 0.12). There was a tendency for hay to contain more hemicellulose (*p* = 0.090). Hay contained more CP than straw (*p* = 0.012). As a comparison, previously reported composition of sedges is also included (Table 1).

### 3.2. Ruminal Fiber Digestion Kinetics

**Figure 1:**
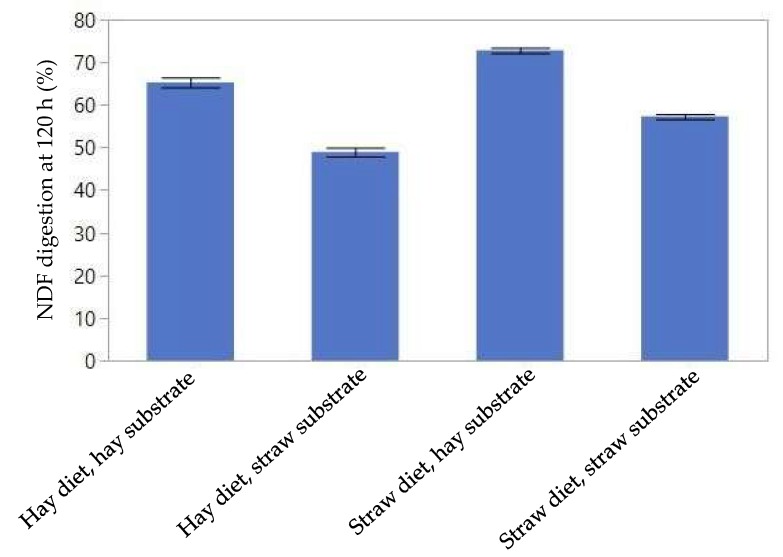

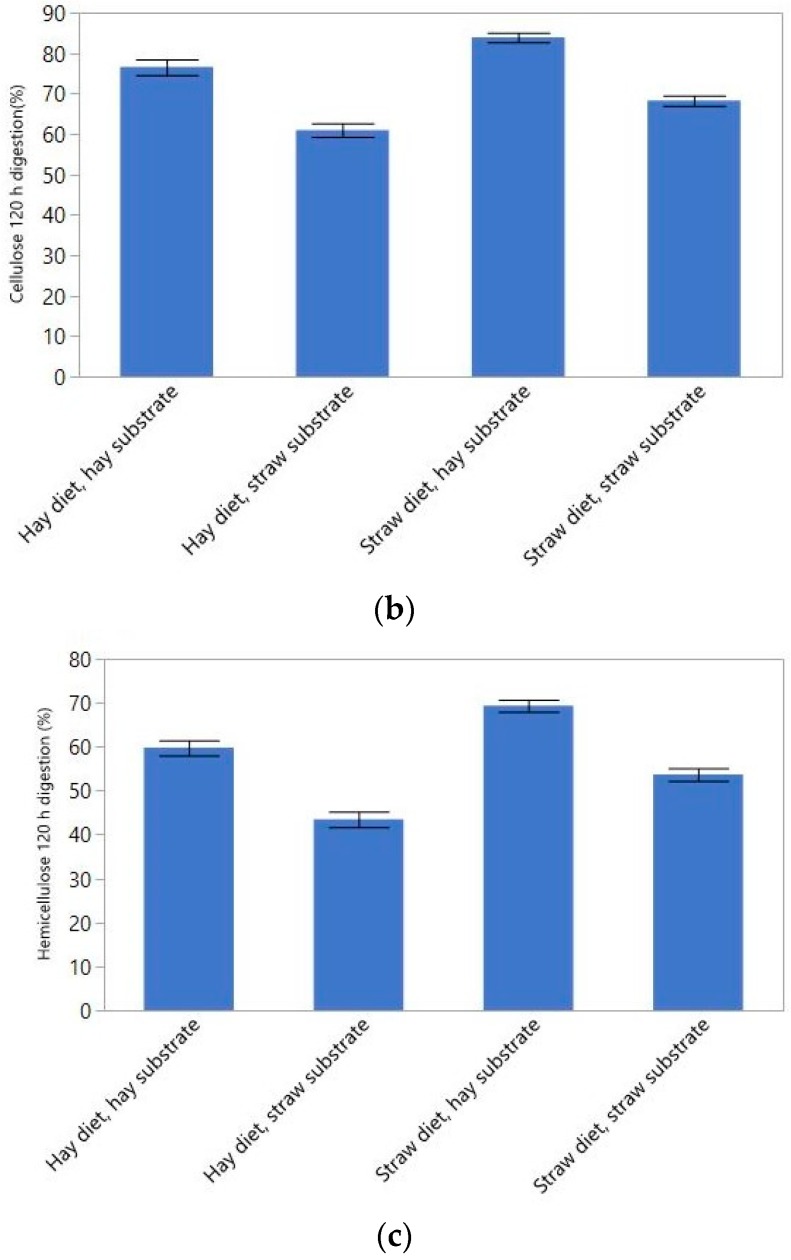
Digestion (%) of NDF (**a**), cellulose (**b**), and hemicellulose (**c**), at 120 h in nylon bags with different substrates and animals on different diets.

Animals digested NDF at a higher rate (0.022 vs. 0.017 h^−1^; *p* < 0.001) and to a greater extent (*p* < 0.001; Figure 1; Supplementary Figures S1 and S2) when the diet was straw rather than hay. Straw NDF was digested faster in the spring than the fall (0.0234 ± 0.0022 vs. 0.0145 ± 0.00231 h^−1^; *p* < 0.001), whereas there was no effect of season (*p* = 0.72) on rate of NDF digestion in hay (interaction season by substrate *p* = 0.012). Extent of digestion of NDF was greater in the hay than in the straw substrate at 120 h (0.678 ± 0.010 vs. 0.540 ± 0.010 kg kg^−1^; *p* < 0.001).

### 3.3. Ruminal pH

Ruminal pH was more alkaline in the spring (6.54 ± 0.04 vs. 6.39 ± 0.04; *p* < 0.001) and tended to be more alkaline in animals eating straw (6.50 ± 0.04 vs. 6.43 ± 0.04; *p* = 0.06). Ruminal pH was positively related to NDF digestion rate (R^2^ = 0.344; *p* < 0.001), 120 h NDF digestion extent (R^2^ = 0. 077; *p* < 0.001), cellulose digestion rate (R^2^ = 0.159; *p* = 0.001), and hemicellulose digestion rate (R^2^ = 0.201; *p* < 0.001). Ruminal pH was weakly related to the extent of hemicellulose digestion at 120 h (R^2^ = 0.0603; *p* = 0.006) but unrelated to that of cellulose (*p* = 0.24).

### 3.4. Bacterial Community Analyses

The number of sequences assembled was on average 36,980, 22,112, 32,619 and 35,607 for the straw diet in the spring, the straw diet in the fall, the hay diet in the spring, and the hay diet in the fall, respectively. Chao’s estimates of sequence richness were 8181 for straw and 9269 for hay in spring, whereas fall sequences were estimated at 13,262, and 14,394 for straw and hay, respectively. Sequence coverage was estimated at 94.2% and 92.7%, respectively, for straw and hay in spring, whereas fall coverage was 85.7% and 89.5% for straw and hay respectively. The Shannon diversity index was consistent between seasons and diet at 6.39 and 6.69 for the straw and hay in spring and at 6.59, and 6.92 for the straw and hay in fall.

Bacterial community structure, in terms of proportional representation of individual phyla, was affected by season (F_10,17_ = 2.82, *p* = 0.029; Table 2) and diet (F_10,17_ = 2.95, *p* = 0.024). Together, Bacteroidetes and Firmicutes accounted for over 90% of sequences with no changes between seasons and diets. Fibrobacteres 16S rRNA gene proportion of total bacterial 16S rRNA gene was greater in animals eating the triticale straw diet (*p* = 0.036).

### 3.5. Association between Fiber Digestion Kinetics and the Proportion of Bacterial Sequences

For both the hay and straw substrates, the greatest percentage of variation in fiber digestion kinetics for all 6 response variables combined (rate and 120 h digestion extent of NDF, cellulose and hemicellulose) was explained by grouping bacterial sequences at the genus level (Table 3).

The proportional representation of bacterial genera in the rumen explained 50 to 70% of the variation in the rate or 120 h extent of digestion of the different fiber fractions in hay (Table 4) and straw (Table 5) as estimated using partial least squares regression. Out of 80 bacterial genera, around 40 were deemed important (variable importance score > 0.8) in fitting the models for digestion kinetics of the different fiber fractions. Some of the genera that had a consistent positive association with fiber digestion rate and/or extent were *Catenibacterium*, *Catabacter*, *Clostridium* and *Papillibacter*. *Catabacter* spp., *Catenibacterium* spp. and *Papillibacter* spp. were all more abundant with the straw diet than with the hay diet; *Clostridium* spp. was more abundant with the straw diet only in the spring (*p* < 0.001), but not in the fall (*p* = 0.90). On the other hand, *Roseburia*, *Prevotella*, *Coprococcus*, *Quinella* and *Pseudobutyrivibrio* were often negatively associated with fiber digestion rate and/or extent (Table 4 and Table 5).

## 4. Discussion

### 4.1. Intake, Digestion and Body Mass Changes

DM intake in the fall was 3.2-fold greater for the hay than for the straw diet. In ruminants eating high-roughage diets, intake is physically limited by distension of the reticulo-rumen, and can be increased by greater digestion rate accelerating ruminal emptying [20]. However, in situ NDF digestion rate in the fall was similar for the hay and straw substrates in animals eating the corresponding diets (0.0145 vs. 0.0175 h^−1^; *p* = 0.44), suggesting that digestion rate did not limit straw intake in the fall. Furthermore, the greater potentially digestible NDF fraction of the hay substrate, along with a similar digestion rate, could result in hay particles being retained for longer because of greater buoyancy over time, while fermentation gases are produced, which would delay passage out of the rumen and thus potentially slow ruminal emptying [20]. However, the in situ incubation determination, in which both forages were finely ground, does not take into account possible differences in the times needed to achieve physical disruption and particle size reduction (e.g., mastication and rumination, initial phases of digestion), which might have been greater for straw. Other factors such as N content and palatability may also explain the greater DM intake of hay compared with straw.

In the fall, overall tract digestibility of hay fiber fractions was greater than straw. In general, in situ fiber digestion rates were similar for the hay and straw substrates, but hay had greater extent of digestion at 120 h. On the other hand, similar DM digestibility between the hay and straw diets can be explained by the fact that the animals on straw consumed less forage, and consequently, a greater proportion of their DM intake originated from the supplement (0.24 ± 0.015 vs. 0.092 ± 0.015 kg kg^−1^; *p* = 0.001), which is more digestible than the forages due to its lesser fiber content [18,21].

Animals eating straw had a greater rate and extent of digestion for all fiber fractions of both substrates. This observation can be interpreted on the basis of Michaelis-Menten kinetics. When animals are not adapted to their diets, fibrolytic activity by microbial enzymes may limit digestion of particles in the rumen. Alternatively, when the microbial community is adapted to diet, particle digestion would be primarily limited by substrate kinetics, i.e., surface on the substrate available for microbial colonization and digestion. The response of in situ digestion to diet indicates that digestion of the materials incubated in situ was limited by enzyme kinetics. However, one could expect that in animals adapted to their diet, microbial fibrolytic enzyme activity would match the surface available for digestion (i.e., digestion being primarily limited by substrate kinetics). The diet would influence ruminal microbiota (i.e., enzyme kinetics) but not in situ substrate surface available for microbial digestion. The response of in situ digestion rate and extent to diet, could be due to one of the substrates incubated being intrinsically more digestible than the diet ingested (i.e., response of hay substrate to straw diet) and to the in situ substrate fine grinding, resulting in a greater surface to volume ratio for microbial colonization and digestion in comparison to average digesta particles (i.e., responses of both straw and hay substrates to straw diet).

In the present study, hay NDF digestion rate in muskoxen was numerically smaller than that reported for cattle in studies that evaluated hay with similar ADF content, whereas straw NDF digestion rate was numerically similar (Table 6). Therefore, greater overall tract digestibility of OM, CP and fiber reported for muskoxen compared to cattle [17] may be the result of longer retention in muskoxen than cattle [44] rather than greater rates of digestion in muskoxen than cattle.

The present study failed to find the summer/early fall body mass recovery reported by other studies with captive muskoxen [19]. However, other studies with non-breeding captive female muskoxen [17,50] also did not find clear seasonal patterns of body mass changes. It seems possible, therefore, that seasonal body mass and intake changes in muskoxen could be influenced by year, location and the loss of body mass in the previous winter [19,21]. Also, the differences in fiber digestion kinetics seasonal patterns between the present study and the study by Barboza et al. [18] suggest that microbial populations probably varied between these two studies. Considering the variation between years and locations in body mass change patterns and digestion kinetics, that different studies report, a more complete characterization of the ruminal microbial population in muskoxen may need replicated experiments at different times, diets and locations.

### 4.2. Bacterial Community

An increase in bacterial richness between spring and fall without major changes in diversity indices may be explained by a decrease in the evenness of sequences. Muskoxen evolved in the high Arctic and remained isolated from domestic ruminants during their evolutionary history, so they could harbor organisms that have not been reported for the rumen of domestic ruminants or other gut environments. Even though these animals had not been in contact with domestic ruminants, they had been raised in captivity for several generations. In agreement, a large proportion of uncharacterized bacteria phylotypes was isolated from muskoxen feces, with a preponderance of Firmicutes and Bacteroidetes [7]. Studying the ruminal microbiota of wild muskoxen could likely further expand the current knowledge of the microbial community in ruminants.

The present analysis of relationships between fiber digestion kinetics and bacterial community structure does not prove causal relationships. Therefore, an association between fiber digestion kinetics and the relative abundance of a bacterial taxon could be the result of: (i) direct involvement of the organism in fiber digestion; (ii) indirect effect of the organism on fiber digestion, be it stimulatory (e.g., providing nutrients to fiber digesters, removing fermentation products) or inhibitory (e.g., producing bacteriocins against fibrolytic organisms); (iii) reverse causal relationship i.e., fiber digestion affecting a particular bacterial group; (iv) association without any direct or indirect effect of the organism on fiber digestion or its reversal.

Because 16S rRNA gene copy numbers and PCR efficiencies vary among ruminal organisms, it should be noted that 16S rRNA gene sequences do not correspond exactly with bacterial numbers [51] or activity. Furthermore, the associations reported herein involve 16S rRNA gene proportions and not absolute gene numbers. Therefore, some of these relationships would need to be further explored with a more quantitative technique such as qPCR or metatranscriptomic analysis.

In the rumen of domestic animals, the bulk of fiber digestion is thought to be carried out by three species of highly specialized cellulolytic bacteria, *Fibrobacter succinogenes*, *Ruminococcus flavefaciens*, and *R. albus* [51]. However, in muskoxen, it was only the genus *Fibrobacter* spp. that appeared within the upper 5th percentile of genera associated with any of the fiber digestion parameters studied, and only in the case of cellulose digestion rate of the straw substrate (Table 4 and Table 5). Furthermore, *Ruminococcus* spp. had a negative association with NDF and cellulose digestion rate in straw. However, not all of the species of the genus *Ruminococcus* are known to be cellulolytic [52]. Instead, organisms other than the typical fiber digesters of domestic ruminants were positively associated with fiber digestion in a consistent way and may be potentially important in muskoxen. *Clostridium* spp., *Catabacter* spp., *Catenibacterium* spp. and *Papillibacter* spp. frequently manifested positive associations with digestion rate and extent of the different fiber fractions both in hay and straw (Table 4 and Table 5). Cellulolytic clostridia inhabiting the rumen have been previously described [53]. However, the genus *Clostridium* is phylogenetically very heterogeneous and contains organisms from diverse phylogenetic groups [54]. Three clostridial species were identified in the present analysis: *C. leptum*, *C. orbiscindens*, and an unidentified clostridial OTU. Of those, only *C. leptum* had a positive association with fiber digestion. This organism belongs to the *Clostridium* Cluster IV, whose 16S rRNA gave numerous signals associated with ruminal particulate fraction when tagged with fluorescent probes [55].

The genus *Catabacter* has been identified in cattle feces [36]. Although its ecological niche in the ruminant gut remains unknown, the inability of *Catabacter hongkongensis*, the only species of *Catabacter* so far isolated, to utilize cellobiose [56] could cast some doubt about a possible direct role of the *Catabacter* spp. found in the present study on cellulose digestion. The genus *Catenibacterium* was also positively associated with fiber digestion. *Catenibacterium mitsuokai* was able to ferment cellobiose [57], although its capacity to digest cellulose was not reported. Although it could utilize mannose, it did not use xylose or arabinose [57], which makes it unlikely to digest hemicellulose.

The proportion of 16S rRNA gene of some genera frequently had a negative association with fiber digestion: *Roseburia* spp., *Coprococcus* spp., *Quinella* spp., *Prevotella* spp. and *Pseudobutyrivibrio* spp. (Table 4 and Table 5). *Roseburia* spp. and *Coprococcus* spp. isolates are butyrate producers in the human gut [58], both genera belonging to *Clostridium* Cluster XIV [54]. *Quinella* spp. was abundant in the rumen of sheep fed high-sugar diets of fresh alfalfa [59]. *Prevotella* spp. does not degrade cellulose, but can use xylans and pectins [53]. The *Butyrivibrio*/*Pseudobutyrivibrio* group is phylogenetically diverse [60], which makes it difficult to draw more accurate conclusions about the possible significance of organisms belonging in this group in fiber digestion in muskoxen.

In summary, with the possible exception of cellulolytic clostridia, there is no clear indication at this point that the associations observed between bacterial 16S rRNA gene proportion and fiber digestion could be the consequence of causal relationships. Moreover, negative association of a cellulolytic genus like *Ruminococcus* spp. [53] or cellulolytic/xylanolytic/pectinolytic-containing species/strains genera like *Roseburia* spp. [61,62] with fiber digestion sometimes occurred. Perhaps these organisms may have inhibited other fiber-digesting organisms. For example, inhibition of fibrolytic anaerobic fungi by *R. flavefaciens* and *R. albus* has been reported [63].

### 4.3. Implications for Muskoxen Biology

Feeding muskoxen a low-quality diet stimulated fiber digestion. These changes in fiber digestion kinetics in response to diet were accompanied by corresponding changes in the microbiome. There was a greater abundance of those organisms most positively associated with fiber digestion when the animals were fed straw rather than hay; therefore, changes in ruminal microbiome that affect digestion appear to be driven by the quality of the forage available. As a limitation to the findings on the association between fiber digestion and changes in the abundance of microbial groups, it must be pointed out that only microbiota in rumen particles, but not fluid, was sequenced, and it has been shown that the bacterial community compositions are distinct [64]. That said, fiber-digesting bacteria, which were the principal object of the microbial diversity analysis conducted, are preferentially attached to solid particles [65].

Barboza et al. [18] found greater fiber digestion rate in the fall compared to spring and winter. In the present study, on the other hand, we found greater fiber digestion rates in the spring compared to the fall, but this was true only for the straw substrate, and there was no difference for hay, which was the same substrate that Barboza et al. [18] used. It appears that, although muskoxen do modify intake and digestion according to season and independently of diet [17,18,19], the effect of dietary changes on intake and digestion may be greater than the effect of season alone. Seasonal changes in food selection that accompany changes in the phenology and abundance of plants may have the greatest influence on microbial function, fiber digestion and mass gain of muskoxen in the wild [66].

Digestion rates of medium- and low-quality forages seemed to be similar or even lower in muskoxen than in cattle (Table 6), and previous results showing greater overall tract digestibility in muskoxen compared to cattle during the spring [18] appear to be due to slow outflow of digesta. Muskoxen have evolved to digest a wide variety of plants, including those consumed by cattle, but also tolerate large changes in availability and quality of those forages through the year. Consequently, they have also co-evolved a unique ruminal microbiome that shares some organisms with cattle and other gut-related environments, but also includes organisms never described for those environments. In agreement, fibrolytic enzymes in eukaryotes inhabiting the muskoxen rumen differ from those found in domestic ruminants [31]. The muskoxen rumen harbors fibrolytic organisms whose novel enzymes are currently being prospected for their potential use in other areas such as biofuel production from lignocelluloses.

## Figures and Tables

**Table 1:** Feed composition.

Feed	DM (kg kg^−1^)	NDF (kg kg DM^−1^)	ADF (kg kg DM^−1^)	Cellulose (kg kg DM^−1^)	Hemicellulose (kg kg DM^−1^)	Lignin (kg kg DM^−1^)	N (kg kg DM^−1^)
**Hay**	0.875	0.700	0.379	0.318	0.322	0.061	0.0756
**Straw**	0.878	0.846	0.536	0.435	0.310	0.10	0.0359
**SEM**	1.82 × 10^−3^	0.0090	0.00649	0.022	0.0077	0.027	4.31 × 10^−3^
***p* =**	0.49	<0.001	<0.001	<0.001	0.090	0.12	0.012
**Sedge**		0.645–0.717	0.284–0.370	0.214–0.291	0.347–0.395	0.0621–0.0797	0.0638–0.156

NOTE: DM = dry matter; NDF = neutral detergent fiber; ADF = acid detergent fiber; N = nitrogen; Information of the sedge proximal composition is from Gustine et al. (2017) [42] and Barboza et al. (2018) [43]. Moisture content of fresh sedges were not measured.

**Table 2:** Effects of season and diet on the proportion of bacterial phyla.

Phylum	Season		Diet		Season *p* =	Diet *p* =	Season × Diet *p* =	SEM
	Spring	Fall	Hay	Straw				
**Actinobacteria**	4.98 × 10^−3^	4.42 × 10^−3^	4.94 × 10^−3^	4.46 × 10^−3^	0.40	0.47	0.25	3.3 × 10^−4^
**Bacteroidetes**	0.335	0.346	0.340	0.341	0.37	0.93	0.35	5.53 × 10^−3^
**Fibrobacteres**	2.81 × 10^−2^	3.42 × 10^−2^	2.46 × 10^−2^	3.77 × 10^−2^	0.32	0.036	0.35	2.96 × 10^−3^
**TM7**	5.65 × 10^−3^	3.85 × 10^−3^	5.88 × 10^−3^	3.62 × 10^−3^	0.005	0.001	0.86	3.1 × 10^−4^
**Firmicutes**	0.577	0.566	0.577	0.566	0.39	0.30	0.10	5.48 × 10^−3^
**Lentisphaerae**	6.13 × 10^−3^	4.73 × 10^−3^	5.50 × 10^−3^	5.36 × 10^−3^	0.14	0.88	0.93	4.8 × 10^−4^
**Planctomycetes**	1.18 × 10^−3^	3.94 × 10^−4^	4.95 × 10^−4^	1.08 × 10^−3^	0.020	0.08	0.16	1.6 × 10^−4^
**Proteobacteria**	1.44 × 10^−2^	1.63 × 10^−2^	1.63 × 10^−2^	1.44 × 10^−2^	0.33	0.29	0.91	8.8 × 10^−4^
**Spirochaetes**	2.13 × 10^−2^	1.55 × 10^−2^	1.70 × 10^−2^	1.98 × 10^−2^	0.016	0.21	0.75	1.10 × 10^−3^
**Synergistetes**	8.1 × 10^−4^	1.12 × 10^−3^	1.10 × 10^−3^	8.27 × 10^−4^	0.41	0.43	0.48	1.7 × 10^−4^

NOTE: SEM = standard error of the mean.

**Table 3:** Percentage of variation in fiber digestion explained by bacterial sequences classified at different clade levels.

Clade	Hay Substrate		Straw Substrate	
	Variation in Response Explained (%)	Latent Vectors	Variation in Response Explained (%)	Latent Vectors
**Phylum**	14.8	2	14.9	1
**Class**	21.4	1	22.5	2
**Order**	20.7	1	25.1	2
**Family**	34.1	1	32.2	3
**Genus**	39.3	1	36.9	2
**Species**	35.1	1	32.7	3

**Table 4:** Main bacterial genera associated with fiber digestion in hay.

	NDF Digestion Rate	NDF Digestion at 120 h	Cellulose Digestion Rate	Cellulose Digestion at 120 h	Hemicellulose Digestion Rate	Hemicellulose Digestion at 120 h
**%response explained**	49.8	69.5	47.7	57.9	49.8	48.4
**Number of important genera (variable importance score > 0.8)**	39	36	42	43	41	37
	**Positive Associations (≥95% Percentile Variable Importance Score)**					
	*Catenibacterium*	*Catenibacterium*	*Clostridium*	*Papillibacter*	*Catabacter*	*Clostridium*
	*Catabacter*	*Clostridium*	*Catenibacterium*	RC9 gut group	*Aquiflexum*	*Barnesiella*
	*Aquiflexum*	*Papillibacter*	*Catabacter*	*Desulfonatrovibrio*	*Hydrogenoanaerobacterium*	*Catenibacterium*
	*Clostridium*	*Catabacter*	*Ruminobacter*	*Catenibacterium*	*Papillibacter*	*Papillibacter*
	**Negative Associations (≥95% Percentile Variable Importance Score)**					
	*Thalassospira*	*Coprococcus*	*Prevotella*	*Thalassospira*	*Kiloniella*	*Kiloniella*
	*Prevotella*	*Ruminococcus*	*Heliobacillus*	*Prevotella*	*Prevotella*	*Quinella*
	*Roseburia*	*Pseudobutyrivibrio*	*Roseburia*	*Bacteroides*	*Roseburia*	*Roseburia*
	*Coprococcus*	*Roseburia*	*Thalassospira*	*Roseburia*	*Coprococcus*	*Pseudobutyrivibrio*

NOTE: NDF = neutral detergent fiber.

**Table 5:** Main bacterial genera associated with fiber digestion in straw.

	NDF Digestion Rate	NDF Digestion at 120 h	Cellulose Digestion Rate	Cellulose Digestion at 120 h	Hemicellulose Digestion Rate	Hemicellulose Digestion at 120 h
**%response explained**	59.6	72.4	49.9	56.3	56.7	56.5
**Number of important genera (variable importance > 0.8)**	41	37	40	39	42	38
	**Positive Associations (≥95% Percentile Variable Importance Score)**					
	*Ruminobacter*	*Catabacter*	*Catabacter*	*Papillibacter*	*Ruminobacter*	*Catabacter*
	*Treponema*	*Catenibacterium*	*Fibrobacter*	RC9 gut group	*Clostridium*	*Barnesiella*
	*Clostridium*	*Papillibacter*	*Ethanoligenens*	*Catenibacterium*	*Treponema*	*Clostridium*
	*Catabacter*	*Clostridium*	*Anaerofustis*	*Aquiflexum*	*Dolosigranulum*	*Catenibacterium*
	**Negative Associations (≥95% Percentile Variable Importance Score)**					
	*Quinella*	*Quinella*	*Ruminococcus*	*Quinella*	*Geosporobacter*	*Kiloniella*
	*Pelospora*	*Pseudobutyrivibrio*	*Coprococcus*	*Prevotella*	*Proxilibacter*	*Pseudobutyrivibrio*
	*Ruminococcus*	*Coprococcus*	*Pelospora*	*Thalassospira*	*Desulfonatrovibrio*	*Coprococcus*
	*Oxobacter*	*Roseburia*	*Zhouia*	*Roseburia*	*Anaerophaga*	*Quinella*

NOTE: NDF = neutral detergent fiber.

**Table 6:** Literature comparison of hay and straw NDF digestion rate.

Reference	Animal	Substrate	ADF (kg kg DM^−1^)	NDF Digestion Rate (h^−1^)
Leventini et al. [45]	Cattle	Brome/orchard grass hay	0.39	0.029
Messman et al. [46]	Cattle	Brome grass	0.39	0.065
Carey et al. [47]	Cattle	Brome hay	0.42	0.032
Huhtanen & Vanhatalo [48]	Cattle	Dried timothy	0.37	0.035
Leupp et al. [49]	Cattle	Brome hay	0.38	0.037
P. S. Barboza (pers. com.)	Musk oxen	Brome hay (spring)	0.34	0.009
P. S. Barboza (pers. com.)	Musk oxen	Brome hay (fall)	0.32	0.027
P. S. Barboza (pers. com.)	Musk oxen	Brome hay (winter)	0.35	0.016
This study (average spring)	Musk oxen	Brome hay	0.37	0.019
This study (average fall)	Musk oxen	Brome hay	0.37	0.018
Leventini et al. [45]	Cattle	Wheat straw	NR	0.022
This study (average spring)	Musk oxen	Triticale straw	0.48	0.023
This study (average fall)	Musk oxen	Triticale straw	0.48	0.015

NOTE: NDF = neutral detergent fiber; ADF = acid detergent fiber.

## Supplementary Materials

The following are available online at http://www.mdpi.com/2076-2607/6/3/89/s1, Figure S1: NDF digestion vs. time: hay substrate, Figure S2: NDF digestion vs. time: straw substrate.
