# Supplementary figures and images for: Influence of Season and Diet on Fiber Digestion and Bacterial Community Structure in the Rumen of Muskoxen (Ovibos moschatus)
Source: Microorganisms. 2018 Aug 20;6(3):89. doi: 10.3390/microorganisms6030089 (PMC6165511; doi:10.3390/microorganisms6030089)

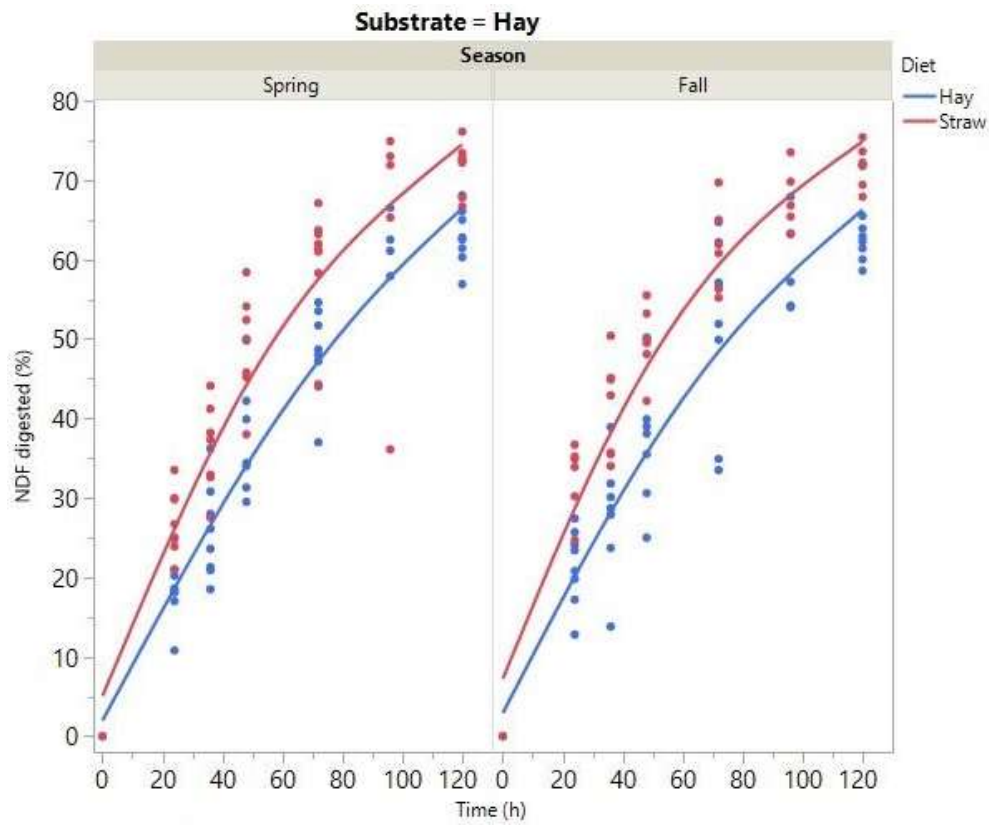

Figure S1. NDF digestion vs. time: hay substrate.

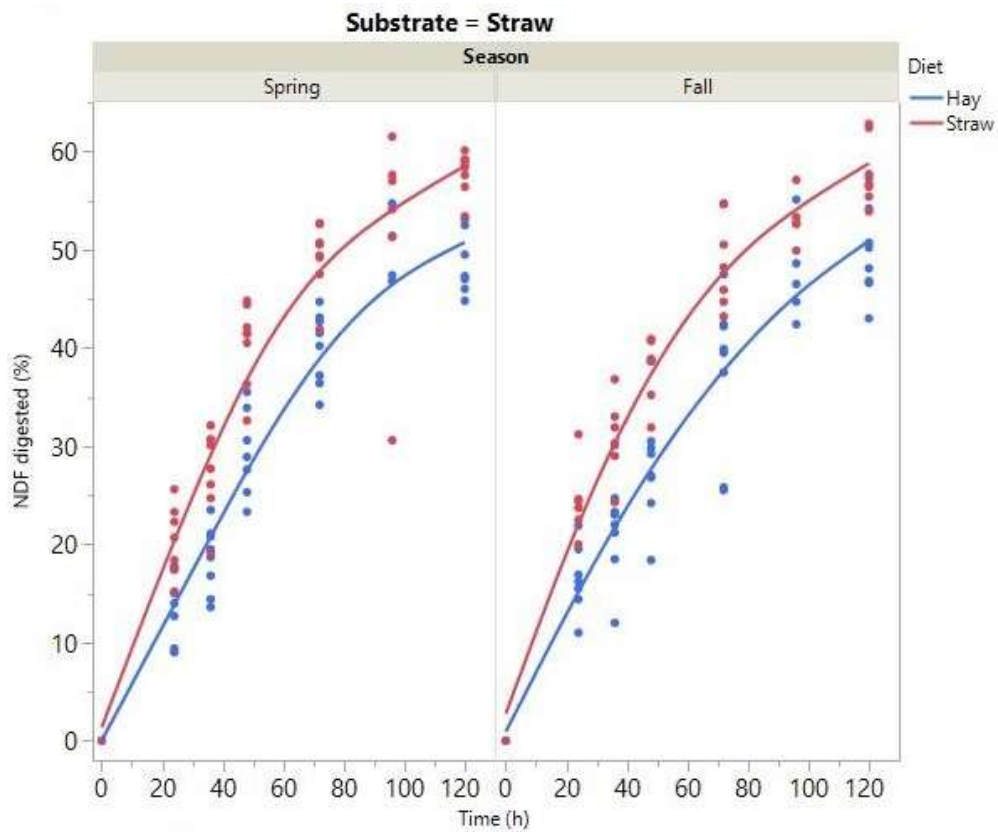

Figure S2. NDF digestion vs. time: straw substrate.

Supplement: Supplementary file 1 [file microorganisms-06-00089-s001.pdf]
